# First detection of the malaria vector *Anopheles arabiensis* in Côte d’Ivoire: urbanization in question

**DOI:** 10.1186/s12936-022-04295-3

**Published:** 2022-09-28

**Authors:** Florence Fournet, Akre M. Adja, Kouassi A. Adou, Milossé M. C. Dahoui, Baba Coulibaly, Konan F. Assouho, Dounin D. Zoh, Moussa Koné, Aboubacar Koné, Koffi L. Niamien, Sylvie Cornelie, Emmanuel Tia, Nicolas Moiroux

**Affiliations:** 1 MIVEGEC (Université de Montpellier, CNRS, IRD), Montpellier, France; 2 Centre d’Entomologie Médicale Et Vétérinaire, Bouaké, Côte d’Ivoire; 3 Institut Pierre Richet, Institut National de Santé Publique, Bouaké, Côte d’Ivoire; 4 Unité de Formation Et de Recherche Biosciences, Université Félix Houphouët-Boigny, Abidjan, Côte d’Ivoire; 5 Institut de Géographie Tropicale, Université Félix Houphouët-Boigny, Abidjan, Côte d’Ivoire

**Keywords:** *Anopheles arabiensis*, Bouaké, Côte d’Ivoire

## Abstract

**Background:**

Previous studies have revealed high malaria transmission in Bouaké, Côte d’Ivoire. The sociopolitical crisis in the country and the resulting environmental changes have raised the need to update existing knowledge on mosquito vector species and malaria transmission.

**Methods:**

Adult mosquitoes were caught using the human landing catch (HLC) sampling method in Bouaké. They were collected in six districts representative of the diversity in urban landscapes. Sampling points were selected along the water network crossing the city and monitored from 2020 to 2021 to detect the presence of anopheline larvae. PCR techniques were used to ascertain the species of the *Anopheles gambiae* complex, *Plasmodium falciparum* sporozoite infection in a subset of *Anopheles* vectors, and insecticide resistance mechanisms in *Anopheles arabiensis* only.

**Results:**

A total of 4599 *Anopheles* mosquitoes were collected and then identified. *Anopheles gambiae *sensu lato (*s.l.)* made up the majority of the whole collection (99%) while *Anopheles funestus* (0.7%), *Anopheles ziemanni* (0.2%), *Anopheles pharoensis* (0.2%) represented the remaining proportion of collection. Among the *Anopheles gambiae* complex, three species were identified namely *An. gambiae *sensu stricto (45.9%), *Anopheles coluzzii* (52.2%), and *Anopheles arabiensis* (1.9%). The first two species had already been collected in Bouaké, however this is the first time that *An. arabiensis* is reported in Côte d’Ivoire. *Anopheles arabiensis* was also collected during the larval surveys in a similar proportion (1.1%) in the same areas as the adults.

**Conclusions:**

This study detected the presence of *An. arabiensis* for the first time in Côte d’Ivoire. This species was found quite far from its usual geographic area and its expansion could be linked to the urbanization process. Although no *An. arabiensis* was found to be infected by *Plasmodium* sp., its role in malaria transmission in Bouaké has to be explored, particularly since its exophagic behaviour raises the issue of control measures and the use of insecticide-impregnated nets. The spread of this species in Côte d'Ivoire should be assessed through further research in additional towns in the country.

## Background

Malaria remains a major public health issue worldwide with 229 million cases and 409,000 deaths reported in 2019 [1]. In the same year, the sub-Saharan African region accounted for 94% of cases and deaths. In Côte d'Ivoire, malaria is the leading cause of medical consultations, with over 5 million cases reported in 2019 [2].

Agricultural development can impact disease dynamics, particularly vector diseases by changing interactions between hosts and vectors as well as by altering vector composition [3]. In the past few years, urbanization has increased worldwide at a fast pace. The United Nations predicts that approximately 70% of the African population will be urban by 2050 [4]. In the early 2000s, medical entomologists thought that urbanization could reduce the burden of malaria because urban environments were considered unsuitable for most major malaria vectors [5, 6]. However, this assumption is now challenged by detailed studies [7]. Indeed, a large variety of *Anopheles* mosquito larval habitats are found in proximity to human dwellings and increase the risk of malaria transmission. These are due to fast-spreading urban or agriculture areas, the presence of standing water, the absence of drainage systems and the poor state of roads linked to unplanned urbanization. In addition, inadequate housing conditions have been recognized as risk factors in urban environments. Donnelly et al. [8] suggested taking these urban factors into account to implement adequate vector control strategies.

In Côte d’Ivoire, 41 *Anopheles* species have been identified to date [9]. Studies carried out in central Côte d’Ivoire by Dossou-Yovo et al. [10] report two major malaria vector species: *Anopheles gambiae *sensu lato (*s.l*.) and *Anopheles funestus*. Assouho et al. [11] showed that *Anopheles nili* is also frequently observed in central Côte d'Ivoire. In the west, Nzeyimana et al. [12] identified two malaria vector species: *An. gambiae *sensu stricto (*s.s*.) and *An. funestus*. According to Adja et al. [13], *An. nili* also plays a major role in malaria transmission in the southern and western forest areas of Côte d'Ivoire. Moreover, *Anopheles coustani, Anopheles ziemanni*, *Anopheles pharoensis*, *Anopheles welcomei*, *Anopheles obscurus* and *Anopheles moucheti* have also been recorded as secondary malaria vectors in the country [10, 14–16].

In Bouaké, the second largest city of Côte d'Ivoire, past and recent studies revealed the main presence of *An. gambiae s.l.* and more specifically *An. gambiae s.s.* and *An. coluzzii*, with *An. funestus*, *An. coustani*, *An. pharoensis*, *An. ziemanni*, *An. obscurus* and *An. welcomei* in low proportions (< 1%) [10, 16].

This study is part of research efforts whose objective is to analyse the impact of the urbanization process on malaria risk factors in Bouaké. The article aims to provide an update on the malaria vector composition in Bouaké.

## Methods

### Study site

The study took place in the city of Bouaké, in the central region of Côte d'Ivoire. The population of Bouaké was estimated to be approximately 540,000 people in 2014 [17]. However, this number is hardly reliable since the census was carried out at the end of a political crisis that occurred in the country between 2002 and 2012. The city spreads out on a plateau and includes a dense hydrographic network that divides in different neighbourhoods and allows both rice cultivation and market gardening in the lowlands. The rainy season runs from April to October, with a decrease in rainfall from July to August. The dry season begins in November and lasts five months. The average annual rainfall is 1200 mm. The average monthly temperatures vary from 24 °C in August to 28 °C in February.

### Mosquito sampling

**Fig. 1:**
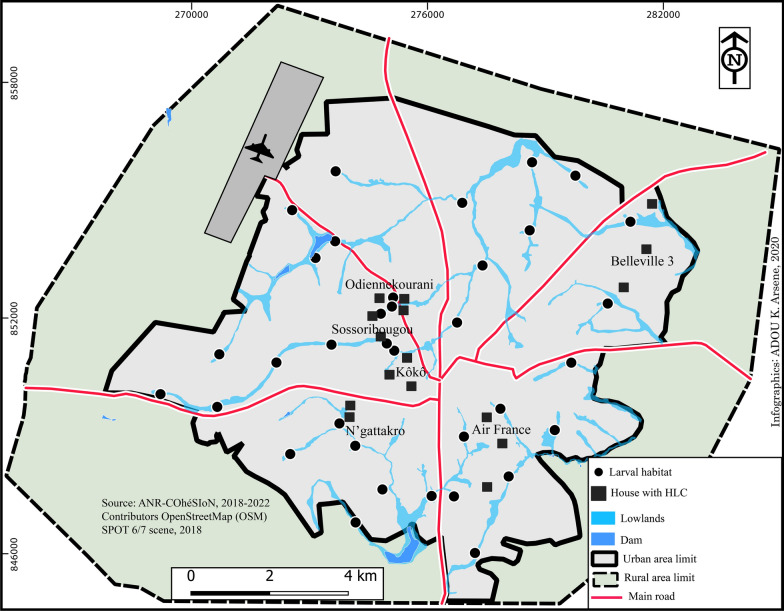
Map of the city of Bouaké showing the sites for the HLC sampling technique and the larval survey

Adult sampling took place in the city of Bouaké, in six districts (Fig. 1), which were representative of the diversity in the urban landscape. Koko is an old central district. Air France is also an ancient urban district, although with higher house standing. Belleville is a recently urbanized district located in the northern part of the town, with a low level of equipment. Sossoribougou district is located near the centre of the city. It is an old irregular district that suffers from a lack of infrastructure. The Odiennekourani and N’Gattakro districts are populated areas along two inland valleys where rice fields and garden markets are intensively developed.

Adult mosquitoes were sampled from 06:00 PM to 08:00 AM, using the HLC technique.

In the districts of Koko, Air France 1 & 2, Belleville 3 and Sossoribougou, sampling was performed at three selected sites per district, both indoors and outdoors, for 2 consecutive nights in March 2019, at the beginning of the rainy season, in October 2019 at the end of the rainy season, and in February 2020 during the dry season (i.e., for a total of 144 nights of HLC sampling).

In Odiennekourani and N’Gattakro, monthly sampling was performed at two sites per district, for only one night, both indoors and outdoors, from February to December 2019, except in April and May 2019 (i.e., for a total of 72 nights of HLC sampling). Two larval surveys were carried out in October 2020 and April 2021. Concentric circles at intervals of 1500 m were plotted over the entire urban area and larval sampling points were selected at the intersections between circles and lowlands. Mosquito larval collections were performed in all potential larval habitats using the dipping method in a 100 m radius at all selected sites. The ecological context (e.g., turbidity, sunlight, type of cultivation, presence of plants and predators) was recorded. The collected larvae were transferred to the insectary of the Institut Pierre Richet (IPR) for rearing.

### Anopheles vector processing

Adult mosquitoes were identified morphologically using the Culicinae identification key of Mattingly [18] and the Anophelinae identification key of Gilles and Coetzee [19]. Only *Anopheles* vectors were individually preserved in Eppendorf tubes (1.5 ml) with silica gel.

All the adults reared from the larval surveys and a random subsample of adult *Anopheles* females from HLC were selected for molecular analysis. DNA extraction was performed from the head and thorax of each specimen following the procedure used by Cornel et al. [20]. The sibling species of the *An. gambiae* complex were identified by PCR using the protocols of Favia et al*.* [21] and Scott et al. [22]. *Anopheles arabiensis* were verified following the protocol described by Fanello et al. [23] to confirm the results. All identified mosquitoes of the *An. gambiae* complex from HLC were tested for the presence of *Plasmodium falciparum* infection using qPCR [24]. Additional PCR assays for the detection of the *kdr west (L1014F), kdr-east* (L1014S) and *ace-1*^*R*^ (G119S) mutations, which confer resistance to insecticides, were performed on *An. arabiensis* specimens collected by HLC according to Bass et al. [25] and Bass et al*.* [26], respectively.

## Results

### Species composition and distribution

A total of 24,125 adult mosquitoes were collected in the six districts. Among these, 73.7% (n = 17,786) were identified as *Culex quinquefasciatus* and 19.1% (n = 4599) were identified as *An. gambiae s.l.* In addition, *An*. *funestus, An. pharoensis and An. ziemanni* were also collected but in very low proportions (< 1%).

Mosquitoes belonging to the *An. gambiae* complex were identified as *An. gambiae s.s*. (n = 1333; 46.0%), *An. coluzzii* (n = 1,517; 52.3%) and *An. arabiensis* (n = 48; 1.7%) (Table 1).

**Table 1:** Distribution of the species within the *Anopheles gambiae* complex collected by HLC in each surveyed district

District	*An. gambiae* s.s N (%)	*An. coluzzii* N (%)	*An. arabiensis* N (%)	Total N (%)
Air France 1 & 2	267 (20.0)	24 (1.6)	0 (0)	291 (10.0)
Belleville 3	272 (20.4)	19 (1.3)	0 (0)	291 (10.0)
Koko	149 (11.2)	182 (12.0)	35 (72.9)	366 (12.6)
Sossoribougou	47 (3.5)	167 (11.0)	1 (2.1)	215 (7.4)
N’Gattakro	371 (27.8)	863 (56.9)	0 (0)	1234 (42.6)
Odiennekourani	228 (17.1)	266 (17.5)	12 (25.0)	506(17.5)
Total	1333 (46.0)	1517 (52.3)	48 (1.7)	2898

**Fig. 2:**
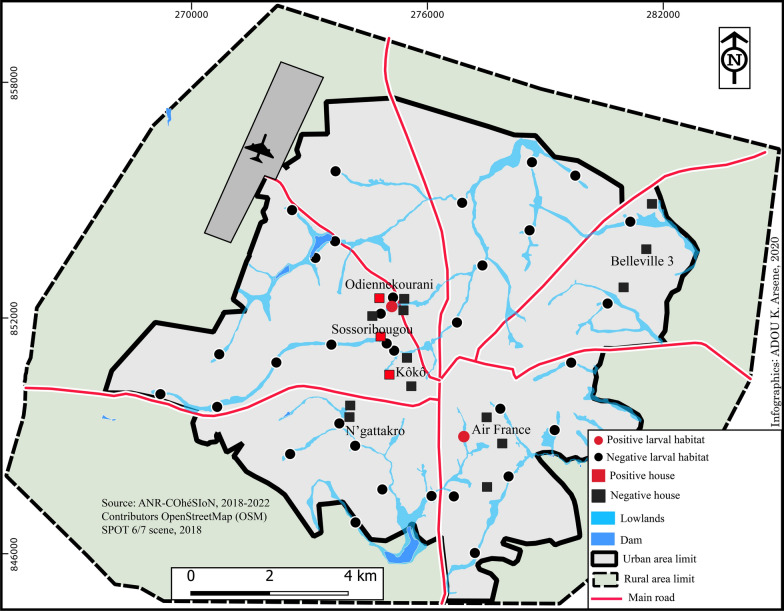
Map of the city of Bouaké showing houses and positive larval habitats to *An. arabiensis*

*An. arabiensis* was found at the end of the rainy season (October 2019), except for one individual captured in March, at the beginning of the rainy season. It was identified only in the districts of Koko, Sossoribougou and Odiennekourani, in the western part of the city, around an inland valley that crosses these districts (Fig. 2).

*Anopheles arabiensis* was only found outdoors (48 individuals caught outdoors, i.e., 0.2 mosquito bite/man/n) and was significantly more active than *An. gambiae s.s.* and *An. coluzzii* during the second part of the night (Chi^2^ = 16.9; p = 0.0019) (Table 2).

**Table 2:** Percentage of *Anopheles gambiae* complex species by time

	*An. arabiensis*	*An. coluzzii*	*An. gambiae* s.s	Total
06:00 PM to 12:00 PM	10.4	26.2	26.7	26.1
12:00 PM to 06:00 AM	87.5	69.8	71.4	70.8
06:00 AM to 08:00 AM	2.1	4.0	1.9	3.0

### Plasmodium falciparum mosquito infection status

None of the 36 mosquitoes of the *An. arabiensis* species tested positive for *P. falciparum* infection. However, 11 (0.73%) *An. coluzzii* and 5 (0.38%) *An. gambiae s.s.* were positive for *P. falciparum* (Table 3)*.*

**Table 3:** *Plasmodium falciparum* infection status of *An. gambiae s.s.* and *An. coluzzii*

	*An. gambiae* s.s		*An. coluzzii*	
	Tested	Infected	Tested	Infected
Air France	267	0	24	0
Belleville	272	0	19	0
Koko	148	1	178	1
Sossoribougou	47	1	167	2
Odiennekourani	228	0	266	1
N'Gattakro	371	3	863	7
	1333	5	1517	11

### Detection of *kdr* mutation in *An. arabiensis*

The *kdr* West and East mutations were detected in 41 (85.4%) and 7 (17.1%) individuals, respectively, revealing an allelic frequency of 1 in both cases. None of the individuals carried the *ace-1*^*R*^ mutation.

### *Anopheles arabiensis* larval habitats

Potential larval habitats for *Anopheles* mosquitoes (i.e., larvae breeding grounds filled with water) were found at 35 and 40 sites (out of the 42 sampled sites) in October 2020 and April 2021, respectively. *Anopheles* larvae were collected in 9 of the 35 sites in October 2020, and then at 24 of the 40 sites in April 2021 (Table 4).

**Table 4:** Different types of the larval habitats detected in Bouaké

	October 2020		April 2021	
	N	Positive n (%)	N	Positive n (%)
Garden market	8	4 (44.4)	20	14 (58.3)
Rice field	9	3 (33.3)	1	0 (0.0)
Puddle	1	1 (11.1)	5	4 (16.7)
Bush	11	0 (0.0)	12	5 (20.8)
Watercourse	6	1 (11.1)	2	1 (4.2)
Total	35	9 (25.7)	40	24 (52.5)

*Anopheles* larvae were found in 33 out of 75 sites (44%). Market gardening accounted for the most positive larval habitat both at the beginning (58.3%) and at the end of the rainy season (44.4%). In the rice fields, 33.3% of the larval habitats were positive at the end of the rainy season. In the early rainy season, positive samples were mostly found in the bush (20.8%) and in puddles (16.7%).

After identification *An. coluzzii* was predominant (60.6%) in the larval sampling (Table 5). Five *An. arabiensis*, and two hybrids of *An. gambiae s.s.* and *An. coluzzii* were found.

**Table 5:** Adult species composition after rearing

	October 2020 N (%)	April 2021 N (%)	Total N (%)
*An. gambiae* s.s	186 (39.6)	66 (34.2)	252 (38.0)
*An. coluzzii*	277 (58.9)	125 (64.8)	402 (60.6)
*An. arabiensis*	5 (1.1)	0	5 (0.8)
*An. obscurus*	2 (0 .4)	0	2 (0.3
*An. gambiae M/S*	0	2 (1.0)	2 (0.3)
Total	470 (70.9)	193 (29.1)	663 (100.0)

**Fig. 3:**
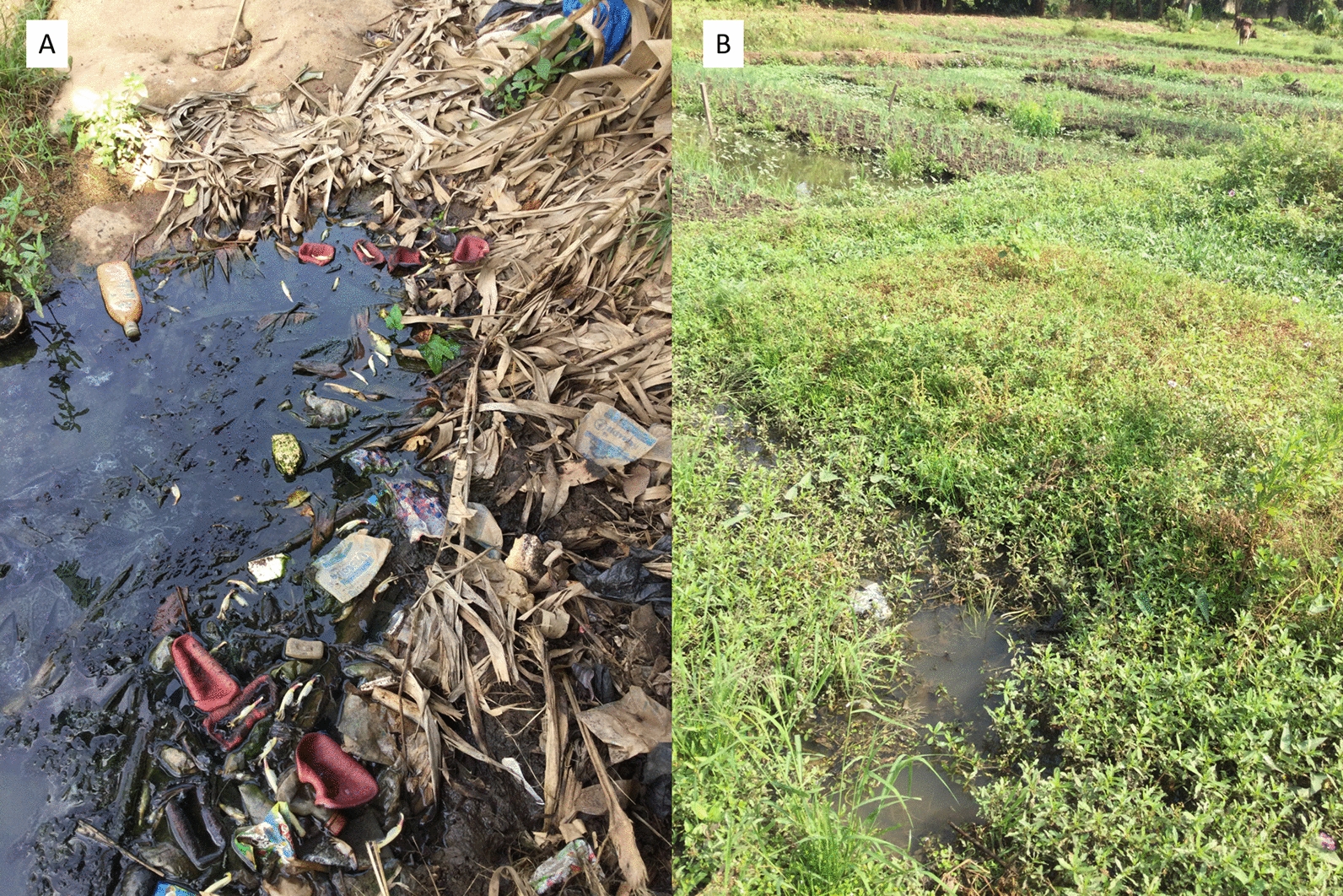
Pictures of the two positive breeding sites for *An. arabiensis* in the city of Bouaké. **A** Breeding site BS36 in a watercourse in the Air France district. **B** Breeding site BS1 in the Odiennekourani district, among old rice fields

*Anopheles arabiensis* individuals were only found at the end of the rainy season in 2020, and at only two sites (Fig. 3). The first site was a watercourse in the Air France district (BS1, Fig. 3B) and the second was located in the lowland of Odiennekourani (BS36, Fig. 3A), near the house where adults were caught in October 2019 .

## Discussion

This study is the first to identify the malaria vector *An. arabiensis* in the city of Bouaké and in Côte d’Ivoire, both at the larval and adult stages. In West Africa, the typical distribution area of *An. arabiensis* was characterized by annual precipitation rates of less than 1000 mm [27, 28]. This area extends from the Atlantic coast in Senegal to the north of Cameroon. Although *An. arabiensis* is mostly found in rural areas, this species has been observed in urban environments to which it has well adapted. In Senegal, it can be found in the capital city, Dakar, where it was collected with *Anopheles melas* and *An. coluzzii* [29, 30].

The presence of *An. arabiensis* in cities has been dependent on its local adaptation to polluted breeding sites [31]. Moreover, environmental changes induced by urbanization (i.e., higher temperatures and lower relative humidity) are also expected to provide favourable environments to *An. arabiensis* outside its historical distribution area. In Bobo-Dioulasso, the second largest city of Burkina Faso, this species, which accounted for 3% of the *Anopheles* population in the 1980s [32], reached 8% in 2002 [33], and then over 90% more recently [34], although these rates vary according to the characteristics of the districts in the city [35]. In Nigeria, in the late 1970s, *An. arabiensis* penetrated the forest belt of the Gulf of Guinea, far from its original distribution area, through urbanized areas as observed by Coluzzii et al. [36]. This fact was confirmed in the 1990s and the 2000s [37, 38].

In Côte d’Ivoire, because of the sociopolitical crisis that occurred from 2000 to 2011, only a few studies have been conducted in the city of Bouaké. Before the 2000s, Dossou-Yovo et al. [10] identified *An. gambiae s.l.* as the main vector in Bouaké according to morphological tools. After the crisis, several studies were conducted in and around Bouaké [16, 39–41]. However, the species identification techniques used in these studies (morphological identification or PCR according to Favia et al*.* [21]) did not allow the identification of *An. arabiensis*. Studies conducted elsewhere in the country during the same period (for example, [11, 13, 42]) have the same limitations.

In this study, *An. arabiensis* individuals were found two years in a row. Livestock trade between Burkina Faso and Côte d'Ivoire is significant. It could be suggested that *An. arabiensis* may have been introduced in Bouaké through trucks carrying cattle from Bobo-Dioulasso, for example, especially since *An. arabiensis* exhibits a strong zoophilic behaviour. Genomic analyses to compare the population profile of *An. arabiensis* in Bouaké with those of the subregion, in particular of Bobo-Dioulasso, may help to understand the colonization process.

The biting behaviour (mostly exophilic) and biting time (in the second part of the night) of *An. arabiensis* collected in Bouaké is consistent with existing data [43, 44]. The first site with *An. arabiensis* larvae was a stream that fit the description of “low-flowing and partially shaded streams” cited by Sinka et al. [43]. It is a known type of breeding site for this species and resembles the Houet River in Bobo-Dioulasso where *An. arabiensis* proliferates [34]. The second larval habitat was in a garden market in an irrigated area. This is also a known type of larval habitat for this species [43], and these sites are very common in Bouaké. Indeed, Bouaké shows extensive lowland areas devoted to agriculture that have historically been associated with malaria transmission [10]. The use of these areas may have increased during the political crisis, when Bouaké was isolated from the rest of the country. There is, therefore, a high risk for *An. arabiensis* to proliferate in Bouaké and to increase malaria transmission even if no *An. arabiensis* were found to be carrying *Plasmodium*. Moreover, urban agriculture is usually associated with a high use of pesticides, which may result in the emergence of resistance in malaria vectors [45]. The *kdr* mutations L1014F and L1014S were both identified in the *An. arabiensis* specimens, but not the *ace-1*^*R*^ mutation as it is observed for this species in Bobo-Dioulasso [34]. The number of individuals analysed was low but it should be noted that the L1014F *kdr* mutation frequency observed recently by Zoh et al. [40] in *An. gambiae s.l.* in Bouaké was in the same range.

## Conclusion

In this study the presence of *An. arabiensis* is reported for the first time in Côte d’Ivoire, in the city of Bouaké. The presence of numerous potential breeding sites for this species in the area, as well as its ability to adapt to urban environments, suggest that *An. arabiensis* may play an important role in maintaining malaria transmission in the city in the future. These results call for entomological surveillance in the cities of Côte d’Ivoire and stress the need for alternative intervention strategies to address the expansion of this species.

## Data Availability

The datasets used and/or analysed during the current study are available from the corresponding author on reasonable request.

## Abbreviations

DIIS: Direction de l’Informatique et de l’Information Sanitaire (Department of Information Technology and Health Information)
*Ace-1*^*R*^: Acetylcholinesterase-1 gene
*Kdr*: Knockdown resistance gene
INS: Institut National de la Statistique
WHO: World Health Organization
HLC: Human landing catch method
